# Patient-provider communication regarding drug costsin Medicare Part D beneficiaries with diabetes: a TRIAD Study

**DOI:** 10.1186/1472-6963-10-164

**Published:** 2010-06-14

**Authors:** Julie A Schmittdiel, Neil Steers, O Kenrik Duru, Susan L Ettner, Arleen F Brown, Vicki Fung, John Hsu, Elaine Quiter, Chien-Wen Tseng, Carol M Mangione

**Affiliations:** 1 Division of Research, Kaiser Permanente, Oakland, CA, USA; 2 Division of General Internal Medicine and Health Services Research, University of California, Los Angeles, CA, USA; 3 Mongan Institute for Health Policy, Massachusetts General Hospital, Boston, MA, USA; 4 Pacific Health Research Institute, Family Medicine and Community Health, Honolulu, HI, USA

## Abstract

**Background:**

Little is known about drug cost communications of Medicare Part D beneficiaries with chronic conditions such as diabetes. The purpose of this study is to assess Medicare Part D beneficiaries with diabetes' levels of communication with physicians regarding prescription drug costs; the perceived importance of these communications; levels of prescription drug switching due to cost; and self-reported cost-related medication non-adherence.

**Methods:**

Data were obtained from a cross-sectional survey (58% response rate) of 1,458 Medicare beneficiaries with diabetes who entered the coverage gap in 2006; adjusted percentages of patients with communication issues were obtained from multivariate regression analyses adjusting for patient demographics and clinical characteristics.

**Results:**

Fewer than half of patients reported discussing the cost of medications with their physicians, while over 75% reported that such communications were important. Forty-eight percent reported their physician had switched to a less expensive medication due to costs. Minorities, females, and older adults had significantly lower levels of communication with their physicians regarding drug costs than white, male, and younger patients respectively. Patients with < $25 K annual household income were more likely than higher income patients to have talked about prescription drug costs with doctors, and to report cost-related non-adherence (27% vs. 17%, p < .001).

**Conclusions:**

Medicare Part D beneficiaries with diabetes who entered the coverage gap have low levels of communication with physicians about drug costs, despite the high perceived importance of such communication. Understanding patient and plan-level characteristics differences in communication and use of cost-cutting strategies can inform interventions to help patients manage prescription drug costs.

## Background

The Medicare Part D outpatient drug coverage benefit, instituted in January 2006, was in many ways a response to rising drug costs for seniors [1-3]. However, the standard Part D benefit contains a coverage "gap" in prescription drug cost coverage. In 2006, a period of no coverage for drug costs began when total drug costs reached $2,250 (after an initial $250 deductible and 25% coinsurance rate up to this point), and continued until patients reach an out-of-pocket maximum of $3,600 [4].

Out-of-pocket expenses comprise up to 20% of health care costs in the U.S. [5]. While physicians can play an important role in reducing out-of-pocket drug cost burden for their Medicare Part D patients [6-8], studies suggest that patients and physicians communicate infrequently about out-of-pocket costs [5,7,9,10] and that this communication occurs less often than either of them would like [5,7,8].

Patients with chronic illnesses such as diabetes may be at particular risk for high drug costs, given their need for multiple medications to treat their diabetes and accompanying comorbid conditions [11-13]; these factors may also make them more liable to experience cost-related medication non-adherence [14]. Despite this increased vulnerability, there is little information on how often Part D beneficiaries with diabetes or other chronic illnesses communicate about drug costs with physicians.

Previous studies suggest that patient characteristics (e.g. race/ethnicity) are associated with how likely patients are to communicate with their physicians about drug costs, and with how much they would like to discuss costs with their providers [5,7,9]. Communication may also potentially vary by the wide variety of Part D plan structures and benefits offered to patients [15], including by whether beneficiaries enroll in Medicare Advantage Prescription Drug Plans (MAPDs) that offer a wide range of health care services along with drug coverage or Prescription Drug Plans (PDPs) that offer drug coverage as a stand-alone benefit. However, this relationship has not been explored. The purpose of this report is to examine drug cost communication levels, prescription drug switching by physicians, and self-reported medication non-adherence among Medicare Part D beneficiaries with diabetes, and to assess how these vary across such characteristics.

## Methods

The Translating Research into Action for Diabetes (TRIAD) study is a multi-center study of diabetes care in managed care settings [16]. Within a subset of health plans participating in TRIAD, a cross-sectional survey was conducted from April-October 2007 to examine the implementation of the Medicare Part D drug benefit among beneficiaries with diabetes. Potential survey respondents were enrolled in one of three types of health insurance products in 2006: 1) for-profit Medicare Advantage Prescription Drug (MAPD) plans within eight states, 2) stand-alone, for-profit Medicare Prescription Drug Plans (PDPs) in the same eight states, and 3) an MAPD product offered by a large, integrated delivery system model HMO in California (IDS MAPD). The multi-state plan is a network-model system offering two different Part D benefit designs. One design had a standard coverage gap between $2,250 in total drug costs and $3,600 in out-of-pocket drug costs, and the other provided generic-only medication coverage during this gap. Beneficiaries could have either of the cost-sharing designs through an MAPD plan or through a stand-alone PDP plan. During the coverage gap, beneficiaries that were enrolled in a plan with generic-only coverage continued to have an $8.50 generic copayment but no coverage for brand name drugs, while those with a complete gap in coverage paid full cost for all drugs including generics. Patients in the IDS-MAPD may have had supplemental gap coverage.

To be eligible for the survey, patients were required to have been continuously enrolled in one of the two MAPD plans from 1/01/05 to 12/31/06, or newly enrolled in the PDP plan between 11/15/05 and 3/01/06, and must have hit the Part D coverage gap in expenditures (i.e. had total 2006 drug expenditures that reached $2,250) by October 1, 2006. Previous work suggests that more than 25% of diabetes patients enter the coverage gap annually [17]. Among eligible patients who were beneficiaries in the participating plans, the survey randomly sampled members who were at least 65 years old, spoke English or Spanish. Drug expenditures were obtained using claims data. Patients who could not provide informed consent or were too ill to participate were excluded. Beneficiaries who were low-income subsidy (LICS) qualifiers were also excluded because their Part D benefit does not include a coverage gap. Potential participants were sent a $10 gift card as an incentive, and offered the option of completing a computer-assisted telephone interview or a written survey.

The survey response rate was 58%. Respondents were not significantly different from non-respondents in terms of gender, number of medications, or geocoded census-track income, and differed less than one year in mean age (data not shown); data are unweighted and did not adjust for non-response.

Survey participants were asked if, during 2006, they 1) thought the issue of prescription drug cost was important enough to raise with their doctor; 2) wanted their doctor to consider the cost to them when choosing medication; and 3) talked with any doctor about the amount they had to pay for prescription drugs. Responses to the first question was given on a 4-point Likert scale, and dichotomized for analysis into 'strongly agree/agree' vs. 'disagree/strongly disagree/don't know,' while responses for the last two questions were given as 'yes/no'. Patients were also asked if their doctor switched any prescriptions to a less expensive medication because of cost, or if they had used any medication less often than the doctor prescribed due to the amount they had to pay in 2006 (responses given as 'yes/no').

Multiple logistic regression models were used to create adjusted percents of patients across demographic and health plan characteristics responding 'strongly agree/agree' or 'yes' to the above questions (dependent variables), adjusting for patient demographics, self-rated health status, and a comorbidity score based on a simple sum of 14 self-reported health conditions obtained through the survey (independent variables). Adjusted percents are calculated by setting all characteristics except the variable of interest to the mean, using the coefficients from the model as multipliers. Since the average number of patients per prescribing physician was very low (mean of 1.5 patients per prescribing physician), models did not adjust for patient clustering within physician. Models also adjusted for month entering the gap, number of unique medications taken during 2006, percentage of medications in the first quarter that were generic, and difference between total and out-of-pocket medication costs in the first quarter (obtained through claims data). Analyses were performed using SAS v9.2.

This study was developed and approved by the Steering Committee of the Translating Research in Action for Diabetes (TRIAD) Study and conducted by researchers in two of TRIAD's Translational Research Centers, and approved by the appropriate Institutional Review Boards.

## Results

A total of 1,458 beneficiaries with diabetes entered the coverage gap in 2006 and responded to the survey. Seventy-four percent of patients were white, and 54% were female. Forty-one percent of patients had an annual household income of less than $25 K (Table 1).

**Table 1:** Demographics of the Study Sample (n = 1458)

	Mean (SD) or %
Age	75.0 (5.8)

65-69 years old (n = 293)	20%

70-74 years old (n = 443)	30%

75-79 years old (n = 415)	29%

80-84 years old (n = 218)	15%

85 plus (n = 89)	6%

	

Race/Ethnicity	

White (n = 982)	74%

Latino (n = 212)	16%

Asian/Pacific Islander (n = 43)	3%

African American (n = 57)	4%

Other (n = 39)	3%

	

Female Gender (n = 793)	54%

	

Education	

< High School (n = 269)	19%

High school graduate/some college (n = 822)	60%

4+ years college (n = 284)	21%

	

Annual Income	

< $25 K (n = 491)	41%

$25-40 K (n = 283)	24%

> $40 K (n = 418)	35%

	

Health Plan	

Integrated Delivery System MAPD (n = 509)	35%

For-Profit MAPD (n = 772)	53%

For-Profit PDP (n = 177)	12%

	

Health Status	

Excellent/Very Good (n = 188)	14%

Good (n = 454)	33%

Fair/Poor (n = 741)	53%

	

Comorbidity score (mean +/- SD) (range = 0-14)	4.9 (2.3)

Number of medications in 2006	14.4 (5.3)

Out-of-pocket med costs in Q1 2006	$359 (262)

Mean % of meds that are generic in Q1 2006	66 (21)

Forty-four percent of patients reported discussing drug costs with their physician in 2006, while 76% reported wanting to have such discussions with physicians and 80% wanted their physician to consider costs when prescribing medications (Table 2). Almost half reported that a physician switched them to a less expensive version of their medication due to cost.

**Table 2:** Patient-Provider Communication Regarding Drug Costs

Patient/Plan Characteristics	Thought issue of drug costs important enough to raise with MD (% strongly agree/agree)	Wants MD to consider cost when choosing drugs (% yes)	Talked with MD about amount paid for drugs (% yes)	MD switched any drug to a less expensive version because of cost (% yes)	Used any drug less often than prescribed because of cost (% yes)
Overall Sample (n = 1458)	76%	80%	44%	47%	22%

					

Adjusted Percents+					

Female	78%	84%*	47%	51%	25%*

Male++	74%	78%	43%	44%	20%

					

White++	76%	81%	48%	49%	23%

Non-White	77%	80%	37%***	43%	23%

					

Age 65-74	81%***	83%	50%**	45%	29%***

Age 75++	70%	79%	41%	51%	16%

					

Income < $25 K	80%*	82%	51%***	47%	27%***

Income $25-40 K	78%	84%	49%***	57%*	24%***

Income > $40 K++	72%	78%	37%	41%	17%

					

Integrated Delivery System MAPD++	66%	77%	36%	33%	21%

For-Profit MAPD	81%***	84%*	50%***	53%***	24%

For-Profit PDP	79%*	81%	51%**	57%**	26%

*p < .05, ** p < .01, *** p < .001

+ From models adjusted for age, gender, income, education, comorbidities, race/ethnicity, difference between total and OOP costs in first quarter (Q1) of 2006, % generic medications in Q1 of 2006, and month of gap entry.

++Referent Group.

After adjusting for other covariates, female patients were more likely than male patients to report they wanted doctors to consider costs when choosing medications (84% vs. 78%, p < .05), and to report they used medications less often than prescribed due to cost (25% vs. 20%, p < .05). Minority patients were less likely than whites to report that they had discussed drug costs with MDs (37% vs. 48%, p < .001). Patients age 65-74 were more likely to discuss prescription drug costs with physicians than those over 75 (50% vs. 41%, p < .001), and also were more likely to report taking less medication due to cost (19% vs. 16%, p < .001). Patients with < $25 K annual household income were more likely to think the issue of drug costs were important enough to raise with their doctor (80%), and also more likely to have talked about prescription drug costs with doctors (51%) than patients with household incomes above $40 K annually. These patients also reported more cost-related non-adherence (27% vs. 17%, p < .01). There were no differences in patient-reported communication, physician drug switching, or cost-related non-adherence by level of patient education (data not shown).

Patients in the IDS MAPD plan were less likely than patients in the other two Part D plans to think the issue of drug costs was important enough to raise with their doctors (66% vs. 81% in for-profit MAPD and 79% in PDP, p < .001), and were also less likely to actually have talked with a doctor about prescription drug costs (36% vs. 50% in for-profit MAPD and 51% in PDP, p < .01). IDS MAPD patients reported slightly lower cost-related non-adherence to medications than patients in the other two plans, although this difference was not significant.

## Discussion

In this study, fewer than half of Medicare Part D beneficiaries with diabetes who hit the coverage gap in 2006 had discussed out-of-pocket prescription drug costs with any physician during that year. This is despite the fact that all of the patients in the study had drug costs sufficient to cause them to reach the prescription drug cost coverage gap in 2006, and 22% reported using medications less often than prescribed due to cost. While fewer than half of patients reported discussing drug costs with physicians, more than three-quarters reported thinking the issue of prescription drug costs was important enough to raise with a doctor.

Forty-seven percent of patients reported that their physician had switched them to a less expensive medication after considering patients' out of pocket costs. This finding suggests that there was room for adjustment to lower cost medications, and that many physicians are amenable to considering medication costs in prescribing. The recent health care reforms to Medicare Part D will continue to require significant medication cost-sharing for patients as changes to the benefit are phased in during the next 10 years, particularly for brand-name drugs [18]. Given that physicians do not always know the level of patient out-of-pocket drug costs [19,20], and may have difficulty identifying patients who have problems paying for prescription drugs [7,21], health plans and medical groups may want to consider organizational level interventions designed to facilitate these switches to lower cost medications.

Patient characteristics such as gender, race/ethnicity, income, and age were found to be related to patient-physician communication regarding prescription drug costs and cost-related non-adherence. Minorities talked with their physicians about drug costs less often than white patients, while women, patients with < $40,000 annual income, and younger beneficiaries were at higher risk for cost-related medication non-adherence. This information that gender, age, income, and race/ethnicity are related to levels of drug cost communication and to self-reported non-adherence could be of help to providers and health plans in identifying patients who may need additional help in initiating such conversations.

Significant differences were observed between patients in MAPD plans offered through a not-for-profit integrated delivery system (IDS-MAPD) and patients in for-profit PDP or MAPD plans. Medicare beneficiaries in the IDS-MAPD were much less likely to either think prescription drug costs were important enough to raise with their doctor or to actually discuss prescription drug costs with their doctor than patients in the other two plans. Despite this, IDS-MAPD patients had lower (although not significantly) levels of cost-related medication non-adherence among the plans. Both patients and providers in the IDS-MAPD may perceive that much of what could be done to reduce prescription costs had already occurred within the current system. Patients and physicians may feel that the managed care system stresses the use of lower cost medications in its formulary and elsewhere [22,23], and that patient out-of-pocket costs are unlikely to be reduced through medication switching.

This study has a number of limitations that should be noted. It is possible that Medicare Part D beneficiaries with diabetes are communicating with other health care providers such as diabetes educators and clinical pharmacists about their prescription drug costs, and that these findings under report overall communication. However, the current survey also asked patients about communication with pharmacists regarding drug costs, and found overall levels to be extremely low across plans. It is also possible that geographic differences may underlie some of the differences seen between the IDS-MAPD, for-profit MAPD, and PDP plans. The IDS-MAPD plan was only offered in California, which has a different health care environment than that of other states [24]. The survey asks about experiences in 2006, but was not administered until 2007; this delay may have biased responses to reflect more recent experiences. Patients report that their MD switched them to a less expensive drug due to cost slightly more often than they report talking to their MD about drug costs (47% vs. 44%); this may be due to factors such as MDs acting without consulting patients, switches initiated by pharmacists, or formulary switches when new generics entered the market that cannot be measured in this study context. The survey sample is comprised mainly of patients in MAPD plans; since the majority of Part D beneficiaries nationally are in PDP plans [25], this may make our findings somewhat less generalizable. Finally, this survey only sampled patients who actually entered the Part D coverage gap; patients who did not enter the gap may have different patterns of provider communication than those in this analysis.

## Conclusions

Medicare Part D beneficiaries with diabetes who hit the coverage gap have relatively low levels of communication with physicians about drug costs, despite the high level of perceived importance of such communication. Understanding patient and plan-level differences in communication can help diabetes educators, physicians, and health plans design and implement strategies to help patients at risk for cost-related non-adherence manage their prescription drug costs.

## Competing interests

The authors declare that they have no competing interest.

## Authors' contributions

JAS and CMM led the analysis. JAS drafted the manuscript. NS performed all data analysis OKD, SLE, AFB, VJ, JH, EQ, CWT, JAS, and CMM developed the survey and contributed to the data analysis plan. All authors read and approved the final manuscript.

## Pre-publication history

The pre-publication history for this paper can be accessed here:

http://www.biomedcentral.com/1472-6963/10/164/prepub
